# Population-Level Prevalence, Bother, and Treatment Behavior for Urinary Incontinence in an Eastern European Country: Findings from the LUTS POLAND Study

**DOI:** 10.3390/jcm10112314

**Published:** 2021-05-26

**Authors:** Mikolaj Przydacz, Marcin Chlosta, Piotr Chlosta

**Affiliations:** Department of Urology, Jagiellonian University Medical College, 30-688 Krakow, Poland; marcin.p.chlosta@gmail.com (M.C.); piotr.chlosta@gmail.com (P.C.)

**Keywords:** Poland, epidemiology, population, urinary incontinence

## Abstract

Objectives: Population-level data are lacking for urinary incontinence (UI) in Central and Eastern European countries. Therefore, the objective of this study was to estimate the prevalence, bother, and behavior regarding treatment for UI in a population-representative group of Polish adults aged ≥ 40 years. Methods: Data for this epidemiological study were derived from the larger LUTS POLAND project, in which a group of adults that typified the Polish population were surveyed, by telephone, about lower urinary tract symptoms. Respondents were classified by age, sex, and place of residence. UI was assessed with a standard protocol and established International Continence Society definitions. Results: The LUTS POLAND survey included 6005 completed interviews. The prevalence of UI was 14.6–25.4%; women reported a greater occurrence compared with men (*p* < 0.001). For both sexes, UI prevalence increased with age. Stress UI was the most common type of UI in women, and urgency UI was the most prevalent in men. We did not find a difference in prevalence between urban and rural areas. Individuals were greatly bothered by UI. For women, mixed UI was the most bothersome, whereas for men, leak for no reason was most annoying. More than half of respondents (51.4–62.3%) who reported UI expressed anxiety about the effect of UI on their quality of life. Nevertheless, only around one third (29.2–38.1%) of respondents with UI sought treatment, most of whom received treatment. Persons from urban and rural areas did not differ in the degrees of treatment seeking and treatment receiving. Conclusion: Urinary incontinence was prevalent and greatly bothersome among Polish adults aged ≥ 40 years. Consequently, UI had detrimental effects on quality of life. Nonetheless, most affected persons did not seek treatment. Therefore, we need to increase population awareness in Poland about UI and available treatment methods, and we need to ensure adequate allocation of government and healthcare system resources.

## 1. Introduction

Urinary incontinence (UI) is a complaint of any involuntary leakage of urine [1,2,3]. UI has strong negative effects on physical and emotional health, including embarrassment, social isolation, impaired occupational functioning, depression, poor self-esteem, and overall diminished quality of life [4].

The prevalence and bother of UI have been estimated by population-based studies worldwide. These investigations revealed that UI may affect more than 40% of adults, a relatively high prevalence despite differences in survey methods, data collection, definition of UI, study population, respondent age, and culture or ethnicity [5,6].

However, there are no reliable data concerning UI in the countries of Central and Eastern Europe. In other words, the prevalence, bother, and treatment preferences of individuals have not been reliably evaluated by the definitions of UI endorsed by the International Continence Society (ICS) in any population-representative investigation for Central or Eastern Europe [1,2,3]. Poland is no exception. No study has been performed in Poland to analyze UI at the general population level, despite an imperative to measure the magnitude of this public health issue, to formulate healthcare policy, and guide clinical practice. Because multiple factors may affect health and health-related behavior (e.g., culture or ethnicity), treatment behavior related to UI in Poland or other Eastern Europe countries may also vary. Further, Poland is characterized by unique demographics (i.e., supra-homogenous ethnicity, ≥99% Caucasian, and ≥95% of residents of Polish identity) [7]. Thus, it is important to compare the Polish population estimates with estimates from less uniform populations [8]. Moreover, many people reside in rural areas of Poland; thus, existing population-level data on UI may not be applicable to Poland because most epidemiological data on UI originate from industrialized areas, including data publicized as being from low- or middle-income countries [9,10,11,12]. Consequently, population estimates for UI exclusively focused on the Polish population are greatly needed because they will expedite the formation of interdisciplinary foundations for national health programs, promote adequate allocation of government and healthcare system resources, and increase public awareness. Therefore, the aim of this study was to evaluate the prevalence, bother, and behavior related to the treatment for UI in a population-representative, geographically inclusive sample of adults aged ≥ 40 years.

## 2. Materials and Methods

The data for this report originate from LUTS POLAND, a large, population-representative, prospective, cross-sectional assessment of lower urinary tract and bladder problems in Polish individuals. Detailed descriptions of the concepts, study design, methodology, and data collection for LUTS POLAND are published [13,14,15]; thus, these items are described only in brief in this paper. LUTS POLAND interviewed a geographically comprehensive (urban and rural) and representative pool of Polish men and women, aged ≥ 40 years. The study revealed that UI was the most bothersome lower urinary tract symptom [13]. Therefore, this report is a more thorough, exclusive analysis of UI. The Jagiellonian University Medical College Ethics Committee approved this study (1072.6120.160.2019). In addition, we registered this study with ClinicalTrials.gov (NCT04121936, accessed on 24 April 2021).

### 2.1. Study Design and Data Collection

We created a representative adult pool from the latest census data and a sample matching technique [7]. For urban and rural areas, we used definitions provided by the Central Statistical Office of Poland (Statistics Poland, Polish: Glowny Urzad Statystyczny, member of the European Statistical System) to obtain adequate representation for these two types of regions: urban areas (cities and towns) including areas located within the administrative boundaries of cities and towns, i.e., areas of urban gminas and cities or towns in urban–rural gminas; rural areas (countryside) including areas remaining outside the administrative boundaries of the cities, which consist of areas of rural gminas and rural parts of urban–rural gminas [16]. Ipsos Poland conducted data collection by computer-assisted telephone interviews [17]. All participants were queried about urgency, stress and mixed UI, and leak for no reason [1,2,3]. The respondents rated the occurrence of UI during the prior month with a Likert-like scale (none, less than 1 in 5 times, less than half the time, about half the time, more than half the time, almost always). In addition, participants rated the bother that accompanied UI (not at all, a little bit, somewhat, quite a bit, a great deal, a very great deal). During the interview, respondents also assessed how bladder problems affected their seeking and receiving treatment, satisfaction with their treatment, the treatment methods that were used, and their quality of life.

### 2.2. Objectives

The primary objective of this study was to investigate the overall prevalence of UI in Poland (any type of involuntary leakage of urine). Researchers who conducted previous large- or small-scale studies of UI in the general population used definitions for UI prevalence that varied widely in the range of occurrence [18,19]. To be able to compare our findings with findings from earlier epidemiological analyses, two definitions of UI prevalence were used: definition I, UI that occurred ‘less than half the time’ or more; definition II, UI that occurred ‘about half the time’ or more. Prior to beginning the survey, we pre-specified the primary objective for this analysis in the statistical plan.

The secondary study objectives were to document sex differences in UI prevalence, the prevalence of specific UI types, the bother of specific UI types (UI rated at least “quite a bit” was considered bothersome), the quality of life, the behavior related to the treatment for UI, and the treatment methods that were used.

### 2.3. Statistics

We used descriptive statistics to summarize the demographic variables and evaluate the initial data. Continuous variables were subjected to the Kruskal–Wallis test, and categorical variables were analyzed by the chi-squared test to evaluate differences in UI prevalence. We used regression analysis to measure differences in UI prevalence regardless of age (a signature risk factor for UI) [20]. A *p* value < 0.05 was considered statistically significant. We used SPSS Statistics software (IBM, version 24.0, Armonk, NY, USA) for all data analyses.

## 3. Results

The LUTS POLAND survey included 6005 respondents from throughout Poland, representative for age, sex, and place of residence (including adequate proportions of urban and rural areas).

### 3.1. The Prevalence of UI

**Figure 1:**
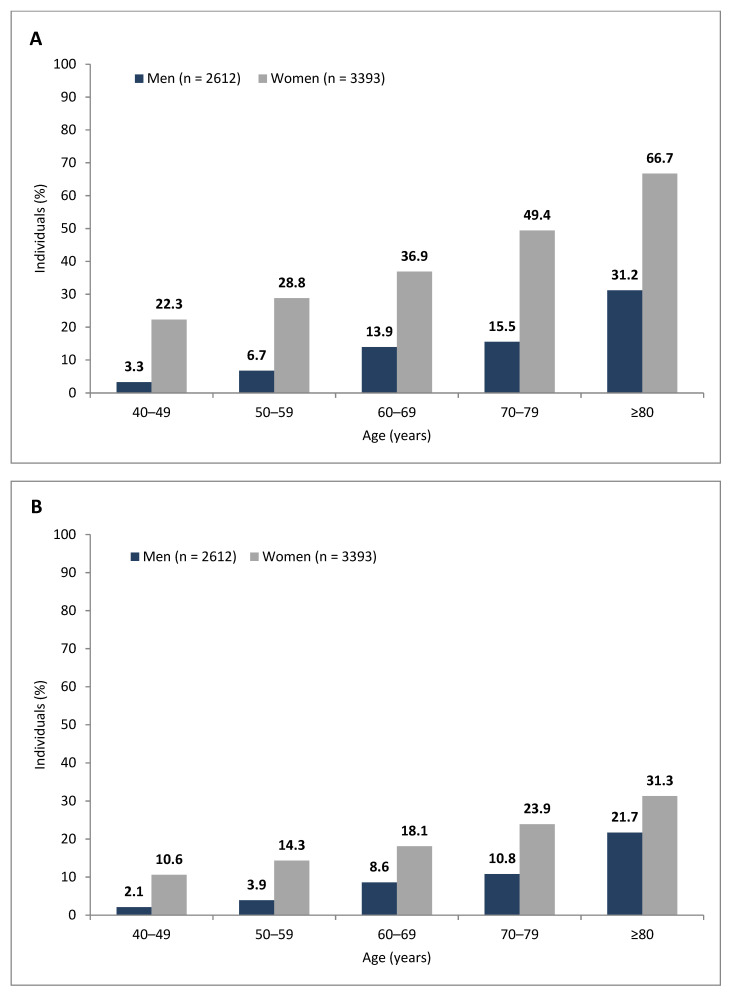
Urinary incontinence prevalence: (**A**) Definition I—urinary incontinence that occurred less than half the time or more; (**B**) Definition II—urinary incontinence that occurred about half the time or more.

The prevalence of UI at least “less than half the time” (definition I) was 25.4% (*n* = 1523); women (*n* = 1242; 36.6%) and men (*n* = 281; 10.8%) exhibited different levels of prevalence (*p* < 0.001). According to definition II, UI at least “about half the time”, the prevalence was 14.6% (*n* = 876); again, the prevalence varied for women (*n* = 723; 21.3%) and men (*n* = 153; 5.9%) (*p* < 0.001). For women and men, the UI prevalence increased in parallel with age (Figure 1). We did not observe urban versus rural differences in UI prevalence. In addition, all provinces (voivodships) of Poland possessed the same UI prevalence.

### 3.2. The Prevalence and Bother of Specific Types of UI

Stress UI was the most prevalent type of UI experienced by women, whereas urgency UI was the dominant UI type in men (Table 1). With both definition I and definition II, we observed these variations in prevalence for different types of UI in women and men.

UI was highly bothersome for women and men. For women, mixed UI was the most bothersome, whereas in a group of men, leak for no reason caused the most bother (not statistically significant observation) (Table 1). There were no geographical or urban/rural differences in the prevalence of UI and bother of specific types of UI.

### 3.3. The Quality of Life

With the question, “If you were to spend the rest of your life with your urinary condition just the way it is now, how would you feel about that?”, we determined that UI had a strong, negative effect on quality of life. On the basis of definition I, we found that 51.4% of the participants with UI responded “mixed”, “mostly dissatisfied”, “unhappy”, or “terrible”. By definition II, we observed that 62.3% of the participants with UI responded similarly. Regardless of the UI prevalence definition, UI had negative effects on quality of life that were comparable between women and men.

### 3.4. Treatment-Related Behavior

According to definition I, less than one third (29.2%, *n* = 445) of the respondents with UI sought treatment, and most of these individuals obtained treatment (24.1%, *n* = 367). With the definition II criterion, slightly more than one third (38.1%, *n* = 334) of the respondents with UI pursued treatment, and most obtained treatment (32.1%, *n* = 281). Men were more active in seeking treatment for UI than women (definition I: *n* = 151, 53.7% of men, *n* = 294, 23.7% of women; definition II: *n* = 96; 62.7% of men, *n* = 238, 32.9% of women; *p* < 0.01). More than half of the participants who reported UI and received treatment were satisfied with their therapy (definition I: 61.3%; definition II: 65.5%). Although men tended to be more satisfied than women with their treatment for UI, this trend was not statistically significant. We did not identify disparities between treatment seeking/receiving/satisfaction, urban/rural areas, and geographical regions (voivodships) of Poland.

Overall, prescription drugs were the most frequently used treatment (definition I: 54.7%; definition II: 64.1%), followed by physiotherapy (definition I: 24.8%; definition II: 33.2%), over-the-counter drugs (definition I: 19.2%; definition II: 24.4%), mechanical devices (definition I: 15.1%; definition II: 23.9%), surgery (definition I: 13.9%; definition II: 23.2%), and lifestyle changes (definition I: 10.3%; definition II: 20.8%). Treatment by two (or more) methods together was adopted by 30.7% (definition I) and 38.1% (definition II) of the participants.

## 4. Discussion

For this investigation, we extracted UI-specific data from the comprehensive, nationwide, and population-representative LUTS POLAND study. Following Russia and Ukraine, Poland has the third largest land mass in Eastern Europe, and Poland is the most eastern country of the European Union. Importantly, this study is the first in Central and Eastern Europe that analyzed, at the population level, the prevalence, bother, and behavior related to the treatment for UI based on a single-country, nationally representative group of adults. The negative effect of UI on public health in this region has been brought into focus by investigations from western countries, which demonstrated that UI in western populations is detrimental for multiple aspects of patient well-being [5,6,21,22]. In addition, populations are becoming increasingly aware of the harmful effects of UI; this recognition further confirms the importance of population-based urological studies in different regions of the world, because concerns exist about inadequate detection and undertreatment for UI [19]. In addition, because our study sample was stratified by age, sex, and place of residence (with sufficient proportions of respondents from urban and rural areas), we further acknowledge the international recommendations that strongly advocate the inclusion of representative pools in studies that analyze UI [23,24]. Importantly, estimates based on representative samples can be generalized more appropriately; therefore, they may more strongly support the governmental and medical systems’ allocation of resources.

Although estimates of the prevalence of UI vary widely [24], UI is a common condition that affects up to 45% of women and 34% of men [6]. A study of adult women from four European countries reported a 35% prevalence of UI, with some variability between the countries [5]. Spain exhibited the lowest prevalence (23%), and France had the highest (43%). In North America, the prevalence of stress, urgency, and mixed UI was reported as 26.8%, 14.4%, and 23.9%, respectively, for women, and 2.9%, 15.2%, and 4.6% for men [6]. In South America, the UI prevalence among Brazilian women was reported to be 52.3% [25]. In Asia, a study of Iranian women estimated the UI prevalence to be 38.4% [12], whereas in Australia, self-reported UI was found in 20.3% of the surveyed population [26]. Our observation of UI in 14.6–25.4% of Poles aged ≥ 40 years, affecting more women (21.3–36.6%) than men (5.9–10.8%), is essentially comparable with findings from the forgoing population-based studies conducted elsewhere. Although it is not completely clear whether UI prevalence varies across racial/ethnic groups (UI prevalence has been suggested to be lower in Hispanic or Afro-American populations [8,27,28,29,30]), UI affects people worldwide, and it is a serious public health concern. Moreover, to some extent, environmental or genetic factors do not appear to affect UI occurrence. Because average life expectancy is increasing in many parts of the world, the global economic burden of UI is projected to further increase and continue to affect public health [31].

Assessment of the aggravation caused by UI is of the utmost importance in population-based studies because an individual’s perspective on bother is more relevant compared with how researchers define UI. In our large cohort, UI was highly bothersome, and it degraded the participants’ quality of life. These results mirrored findings from other studies in which participants generally identified different types of UI as being the most bothersome [18]. Notably, more than half of the respondents with UI in our study, regardless of the definition of UI prevalence, had concerns about quality of life related to their urinary functioning.

However, despite these concerns, only one third of respondents with UI (29.2–38.1%) sought treatment. Low treatment seeking for UI has been documented in some earlier analyses; therefore, lack of treatment seeking is a significant epidemiological alarm [11,32]. Moreover, help-seeking behavior for UI does not seem to have increased during the preceding few decades [33]. Investigators have offered many explanations for the apparent reluctance to seek treatment for UI. UI is often dismissed without adequate management because of either social stigma and embarrassment or assumptions that UI is a natural consequence of age. In addition, some patients have concerns about the financial costs or the adverse effects of treatment. Cultural issues were also described as determinants for not seeking medical attention [34]. Further, in our study, we learned that men outnumbered women in seeking treatment for UI; based on qualitative research, this trend existed most likely because men fear that their urinary problems reflect serious illnesses such as cancer [35]. Conversely, women tend to associate urinary symptoms with non-oncological conditions such as a urinary tract infection; thus, women may be less likely to comment on their symptoms during routine clinical visits. Therefore, there appears to be a continuing need to educate the public about UI in Poland. The lack of knowledge of treatment options may present barriers to healthcare seeking. Without adequate information, individuals cannot develop optimal treatment-related behavior. Importantly, there is an ample source of education and counselling for UI provided by specialists (e.g., urologists, gynecologists, geriatrists), general practitioners, nurses, and, sometimes, physiotherapists focused on pelvic floor physiotherapy. Thus, what remains is for adequate education to reach the different types of medical professionals, particularly primary care physicians who support the greater part of the population.

In our study, prescribed drugs were the most recommended treatment, and around one third (definition I: 30.7%; definition II: 38.1%) used combined treatment. Curiously, basic management by lifestyle changes was the least common treatment method. This paucity of behavioral approaches is a particular concern because the European Association of Urology currently recommends such therapies, along with patient education, as first-choice treatment options for stress, urgency, and mixed UI [23]. Because lifestyle management is noninvasive and reversible, all patients who desire treatment should begin by adopting a personally agreeable form of change. Moreover, lifestyle changes and behavioral therapies are combined easily with other UI treatments. Thus, adjustment of life activities should be part of any treatment plan. It is possible that these treatment methods are ignored because they require a significant amount of time and effort, and commitment by the patient, with regular follow-up, to achieve success [36].

This study was limited by the fact that persons self-reported UI and treatment for UI. Personal interviews are known to contribute to the underreporting of sensitive issues, such as UI. Cold-callings also particularly limit the precise assessment of UI because some respondents might have considered other lower urinary tract symptoms (particularly postmicturition dribble) as episodes of UI. In addition, we did not ask about concomitant conditions that may lead to or exacerbate UI (e.g., diabetes, heart failure, hypertension, pelvic organ prolapse, and obesity). Without medical confirmation, we would have had considerable difficulty in reliably confirming concomitant conditions from a self-reporting participant interviewed by telephone [6]. Coyne et al. described this limitation of population-based self-reported data [37]. We confined our investigation to a pool of adults aged ≥ 40 years; thus, younger representatives were not addressed. Nonetheless, most large and international population-based surveys of lower urinary tract symptoms included respondents of ≥40 years of age [18,38,39]. The survey methodology was designed in such a way that we could broadly compare our results with those of other populations. In addition, some previous population investigations showed that lower urinary tract symptoms were less prevalent (i.e., 5% or less) in younger age groups [40]. Lastly, we did not seek information about obstacles to obtaining healthcare or about drug-related adverse effects.

## 5. Conclusions

We describe the first countrywide, population-representative epidemiological study of UI in an Eastern European country. UI was a prevalent condition among Polish adults aged ≥ 40 years, and more women than men experienced UI. UI was often troublesome, and UI diminished the quality of life. Nevertheless, most affected individuals did not seek treatment. Although our findings were similar to other epidemiologic studies of UI conducted in different regions of the world, we need to develop strategies to increase the awareness of the population about UI, specifically in Poland.

## Figures and Tables

**Table 1:** Prevalence of different types of urinary incontinence in women and men.

	Urgency Urinary Incontinence	Stress Urinary Incontinence	Mixed Urinary Incontinence ^b^	Leak for No Reason
Women (*n* = 3393)				
Prevalence based on definition I (*n*, %)	316, 9.3% ***	415, 12.2% ***	348, 10.3% ***	163, 4.8% **
Prevalence based on definition II (*n*, %)	181, 5.3% ***	248, 7.3% ***	202, 6.0% ***	92, 2.7% **
Prevalence of bother (at least quite a bit) ^a^ (*n*, %)	161, 88.9%	231, 93.1%	193, 95.6% *	81, 88.0%
Men (*n* = 2612)				
Prevalence based on definition I (*n*, %)	109, 4.2%	53, 2.0%	55, 2.1%	64, 2.5%
Prevalence based on definition II (*n*, %)	61, 2.3%	31, 1.2%	30, 1.1%	31, 1.2%
Prevalence of bother (at least quite a bit) ^a^ (*n*, %)	48, 78.7%	26, 83.9%	26, 78.8%	28, 90.3%

^a^ Bother was analyzed according to definition II. ^b^ Mixed urinary incontinence refers to persons who reported both urgency and stress urinary incontinence symptoms. * *p* ≤ 0.05, women vs. men. ** *p* ≤ 0.01, women vs. men. *** *p* ≤ 0.001, women vs. men.

## Data Availability

Data is contained within the article.
